# Effect of Electromagnetic Field Therapy and Customized Foot Insole on Peripheral Circulation and Ankle–Brachial Pressure Index in Patients with Diabetic Foot Ulcer: A Randomized Controlled Clinical Trial

**DOI:** 10.3390/healthcare14060796

**Published:** 2026-03-20

**Authors:** Mshari Alghadier, Ibrahim Ismail Abuzaid, Hany M. Elgohary

**Affiliations:** 1 Department of Health and Rehabilitation Sciences, Prince Sattam bin Abdulaziz University, Alkharj 11942, Saudi Arabia; 2 Department of Cardiovascular/Respiratory Disorder and Geriatrics, Faculty of Applied Medical Sciences, Jerash University, Jerash 26110, Jordan; ibrahim_ismail_2011@svu.edu.eg; 3 Faculty of Physical Therapy, Qena University, Qena 83523, Egypt; 4 Department of Physical Therapy for Surgery, Faculty of Applied Medical Sciences, Jerash University, Jerash 26110, Jordan; hmielgohary@gmail.com; 5 Faculty of Physical Therapy, Cairo University, Cairo 12613, Egypt

**Keywords:** diabetic foot ulcer, electromagnetic field therapy, silicone gel insole, microcirculation, ankle-brachial index, toe-brachial index

## Abstract

**Background:** Diabetic foot ulcers (DFUs) are considered a prevalent complication of diabetes mellitus, frequently accompanied with compromised peripheral circulation, slower healing, as well as high risk of infection in addition to risk of amputation. Additional treatments that enhance microvascular perfusion and lessen plantar pressure may accelerate the healing process. This study was carried out to examine the impact of pulsed electromagnetic field (EMF) therapy as well as customized silicone gel insoles in terms of peripheral circulation in addition to vascular indices in patients with DFUs. **Methods:** A randomized, controlled clinical trial, including sixty-six adults diagnosed with type II diabetes as well as plantar DFUs (Wagner grade I–II) were divided into three groups (*n* = 22 each): Group A was given low-frequency electromagnetic field therapy (15–50 Hz, 2–5 mT, 30 min, three times per week for 8 weeks), Group B was given a customized silicone gel insoles produced for ulcer offloading, and Group C (control) was given conventional physiotherapy along with wound care. Peripheral microcirculation as well as tissue perfusion were the primary outcomes, and they were measured using Laser Doppler Flowmetry (LDF), Photoplethysmography (PPG), in addition to the Toe–Brachial Index (TBI). The secondary outcome included the Ankle–Brachial Pressure Index (ABPI). A blinded assessor measured the outcomes at the beginning of the study, after the intervention (week 8), and again after the follow-up (week 16). **Results:** EMF therapy significantly improved LDF (baseline: 45.2 ± 6.5 PU; week 8: 62.5 ± 7.2 PU), PPG (0.42 ± 0.08 mV to 0.68 ± 0.10 mV), TBI (0.64 ± 0.07 to 0.82 ± 0.08), and ABPI (0.88 ± 0.06 to 0.97 ± 0.05) compared with insoles and controls (*p* < 0.001, partial η^2^ 0.25–0.37). The insole group exhibited moderate enhancements, whereas the control group demonstrated minor changes. Between-group analyses showed substantial differences in favor of EMF therapy across all measured variables (F = 13.5–19.9, *p* < 0.001). Improvements continued at the 8-week follow-up. **Conclusions:** Patients with DFUs who receive EMF therapy experience a significant improvement in their peripheral microcirculation, tissue perfusion, as well as vascular indices. This is more effective than just mechanical offloading, and custom insoles offer extra benefits by redistributing pressure. Combining EMF therapy with regular DFU care may speed up healing and lower the risk of problems. Additional research should investigate the efficacy of combined EMF as well as off-loading interventions and their long-term outcomes.

## 1. Introduction

Diabetic foot ulcers (DFUs) are a frequent and serious complication among those with diabetes [1]. About 15–25% of people with diabetes will have them at a certain time in their lives [2]. DFUs are linked to high rates of disease, a higher chance of getting an infection, lower extremity amputation, along with elevated healthcare costs [3]. The occurrence of DFUs is attributable to multiple factors, including peripheral neuropathy, peripheral arterial disease, compromised microcirculation, as well as biomechanical stress [4]. Neuropathy reduces protective sensation, and along with vascular insufficiency decreases tissue perfusion and oxygenation, which slows down healing [2].

Peripheral microcirculation is very important for healing wounds. Diabetic patients with poor capillary perfusion have difficulty getting oxygen and nutrients to their tissues. This makes ulcers last longer and raises the risk of infection [5]. Blood glucose control, wound cleaning, sterile dressing, infection control, and offloading are all part of traditional care for DFUs. These steps are important, but the healing rates are still not good enough, and extra treatments are needed to improve blood flow and vascular health [6].

Many people use custom insoles to change the pressure on their feet, lower the risk of repeated injuries, and prevent ulcer progression along with recurrence. Silicone gel insoles via “windows” that offload the pressure of ulcers offer targeted pressure relief and have been demonstrated to improve blood flow in the area by reducing mechanical stress [7,8]. Insoles are effective for redistributing pressure, but they mostly deal with mechanical issues and do not directly enhance microvascular function. Patients with significant vascular impairment or neuropathy may continue to experience slower healing even with optimal offloading [9].

Pulsed electromagnetic field (PEMF) therapy represents a non-invasive treatment that has been shown to be beneficial with tissue repair, angiogenesis, as well as microcirculation. Electromagnetic field (EMF) has been demonstrated to regulate endothelial function, activate fibroblast activity, and promote nitric oxide-mediated vasodilation, which may promote wound healing in diabetic along with ischemic tissues [10,11]. It has many therapeutic effects, including improving the function of endothelial and capillary cells, reducing inflammation, bioactivating cells, and neuromodulation. All of these mechanisms may contribute to improving blood circulation, oxygen status, and tissue regeneration in DFUs [12,13].

Clinical, in addition to preclinical assessments, demonstrate that EMF therapy accelerates ulcer healing, alleviates edema, and improves tissue oxygenation [14]. Nonetheless, data are still restricted, and studies vary in treatment frequency and intensity, along with patient demographics, suggesting the necessity for additional high-quality randomized clinical trials (RCTs) [15]. In theory, the addition of EMF therapy with mechanical offloading may focus on both biological and mechanical aspects that contribute to the pathophysiology of DFUs. Off-loading reduces localized stress, while EMF enhances perfusion along with endothelial function, which probably produces a synergistic influence on ulcer healing [16].

Objective assessments of microcirculation, such as Laser Doppler Flowmetry (LDF), Photoplethysmography (PPG), in addition to the Toe–Brachial Index (TBI), produce information concerning the vascular condition of the injured foot and may act as prognostic factors for healing potential [17,18]. Although initial evidence is promising, there is a deficiency of RCTs that compare EMF therapy with customized insoles or traditional treatments in patients with DFU. Moreover, there is a lack of studies that have quantitatively examined microcirculatory alterations resulting from these treatments by restricting clinical applicability.

As a result of the high prevalence of DFUs, inadequate healing outcomes, and the potential mechanisms of EMF therapy, it is essential to investigate its effectiveness when used alone, or when combined with mechanical off-loading. Comprehending vascular along with perfusion responses may improve treatment protocols and reduce complication rates [19]. The aim of the current study is to compare the impacts of EMF therapy and custom silicone gel insoles, in addition to conventional physiotherapy in terms of vascular indices and tissue perfusion, along with peripheral microcirculation in patients having plantar DFUs. The primary outcomes involved LDF and PPG, whereas the secondary outcomes involved TBI and Ankle–Brachial Pressure Index (ABPI). Our hypothesis was that EMF therapy would significantly improve microvascular perfusion along with vascular indices in relation to insoles and conventional care, whereas insoles would produce moderate enhancements using mechanical off-loading. These findings may inform clinical strategies for optimizing ulcer healing in high-risk diabetic populations.

## 2. Materials and Methods

### 2.1. Study Design

The study used a three-arm parallel-group randomized controlled design to permit a head-to-head comparison of the EMF therapy with customized foot insole intervention and standard care to assess the relative and combined efficacy of these treatment modalities on the peripheral circulation and ABPI in diabetic foot ulcer patients. The intervention time was 8 weeks because past clinical trials have shown that adjunctive physiotherapy that address microcirculation and tissue repair in DFUs can generate detectable changes in peripheral perfusion and wound healing in 6–8 weeks of treatment [16,20]. This period was thus deemed adequate to identify clinically significant changes in peripheral circulation and ABPI whilst ensuring that the participants adhered to the protocol and that the number of dropouts was reduced. This trial was planned, implemented and reported following the Consolidated Standards of Reporting Trials (CONSORT) requirements of RCTs. Every element of the study design, flow of participants, randomization, blinding, outcome measurement, and statistical analysis were presented and reported in accordance with the CONSORT principles of parallel-group RCTs, which guarantees transparency, reproducibility, and compliance with the established reporting standards [21]. The study was registered at ClinicaTrials.gov (NCT07070544) and was approved by the Institutional Research Ethics Committee of the Faculty of Physical Therapy, Badr University in Cairo (Approval No. IRB00014233-53). Informed consent was provided by writing to all the participants before they were enrolled in the study. The participants were provided with both oral and written explanations of the study procedures, possible risks and benefits, and their right to withdraw at any time without consequences. All participants signed written informed consent prior to enrollment.

### 2.2. Participants and Eligibility Criteria

Patients aged 40 to 70 years with a confirmed diagnosis of type 2 diabetes mellitus for more than five years were screened for eligibility. Participants were included if they presented with a plantar DFU classified as Wagner grade I or II [22]. The Wagner classification is commonly used to grade DFUs. Grade I refers to a superficial ulcer limited to the skin layers without involvement of deeper tissues, bone, or infection. Grade II indicates a deeper ulcer that extends into subcutaneous tissue, tendon, ligament, muscle, or joint capsule, but without bone involvement, abscess, or gangrene; with an ulcer duration greater than four weeks, an ABPI ranging from 0.6 to 1.1, the ability to ambulate independently with or without a cane, and stable medical therapy for at least three months before enrollment. Patients were excluded if they demonstrated critical limb ischemia (ABPI < 0.5), infected ulcers, gangrene, or osteomyelitis, as well as those with Charcot foot deformity, severe peripheral vascular disease requiring surgical intervention, or contraindications to electromagnetic field therapy such as pacemakers or metallic implants. Patients were also excluded if they had severe neuropathy resulting in a total loss of protective sensation, foot ulcers necessitating surgical off-loading or instant surgical debridement, and cognitive disorders potentially affecting treatment adherence or participation.

### 2.3. Setting of the Study

This study was performed at the Outpatient Department of the Faculty of Physical Therapy, Cairo University, which has advanced wound assessment tools and a location for supervised physiotherapy along with EMF therapy sessions. All treatments were given in a controlled clinical setting by licensed physiotherapists and qualified research staff. This ensured that the treatments were given in a consistent way and that the staff kept an eye on the patients’ safety, adherence, and ulcer progress. During the study period, regular wound care and patient education were given based on the rules of the institution.

### 2.4. Randomization and Allocation

Utilizing a computer-generated block randomization pattern with a block size of six, suitable subjects were randomly put into one of three groups in a 1:1:1 ratio. Group A received EMF, Group B received a customized silicone off-loading insole, while Group C received control, which included conventional physiotherapy as well as standard wound care. Sequentially numbered, sealed, opaque envelopes with allocation concealment were prepared by an independent researcher that did not participate in the recruitment of the participants, the administration of the intervention or the assessment of the outcomes. The group assignment was placed in each envelope which was opened in turn after the participant was enrolled in the study. The random allocation list was created by another researcher who did not participate in the recruitment, delivery of the intervention, or the evaluation of outcomes, which is why the group assignments were not disclosed and the process of randomization was objective. In order to decrease detection bias, the researcher performing all the measurements was not aware of the group assignment. Participants were told that numerous therapeutic modalities were under assessment, yet they stayed unaware of comparative hypotheses to maintain a single-blind study design. Blinding was ensured by instructing participants not to reveal the specifics of their intervention during the assessment and conduction of standardized procedures to ensure the integrity of the blinding during data collection.

### 2.5. Sample Size Calculation

The sample size estimation was determined according to the principal outcome of peripheral microcirculation, assessed by Laser Doppler Flowmetry (LDF, perfusion units, PU). Laser Doppler Flowmetry was chosen as the main outcome measure to calculate sample size since it is a validated, reliable, and sensitive procedure to measure blood flow in the microvascularity and past research has noted it to be able to measure clinically significant changes in the peripheral circulation of DFU patients [23]. Prior studies involving patients with DFUs suggest an anticipated mean difference of around 15 PU among EMF therapy and the control group, accompanied by a standard deviation of 10 PU [24]. We used a one-way ANOVA for all three independent groups to determine the effect size (f) from Cohen’s d (d = 1.5, f = 0.75). We set the alpha level at 0.05 (two-tailed). To obtain a statistical power of 80% and a significance level (0.05), which is commonly used in clinical trials as a sufficient balance between the likelihood of observing clinically important differences and the feasibility of the study, the sample size was determined [25]. The G*Power 3.1.9.7 calculation showed that each group needed 18 participants. To make up for a possible 15% dropout rate, the sample size was raised to 22 individuals per group, for an overall of 66 individuals participating across the three study groups.

### 2.6. Outcome Measures

A blinded assessor evaluated all outcomes at three intervals: baseline, immediately following the 8-week intervention, as well as at an 8-week follow-up upon the intervention completion to determine the continuation of the treatment effects on the peripheral circulation and the index of ankle and brachial pressure and to assess the long-term sustainability of the therapeutic outcomes. The differences between the EMF therapy, customized foot insoles, and the standard care were perceivable, which is why the participants could not be completely blinded by the treatment modality. To reduce expectation bias, the participants were not aware of the hypotheses and anticipated results of the study. The primary outcomes had been peripheral microcirculation and tissue perfusion. The LDF was used for measuring perfusion units within the ulcer margin, PPG was used for measuring toe blood flow amplitude in millivolts, and the TBI was defined as the ratio of toe systolic pressure to brachial systolic pressure. The ABPI was the second outcome and it was assessed using a handheld Doppler probe and was the maximum ankle pressure (dorsalis pedis or posterior tibial), and it was divided by the brachial systolic pressure. To make sure that individuals were following the interventions, attendance logs for EMF therapy, regular insole use diaries, and weekly phone calls were all used. The ulcer site was also checked every week to make sure it was tolerable and to find any possible problems.

The participants were observed during the intervention and follow-up period regarding any adverse events or side effects concerning EMF therapy, customized foot insoles, or medical care. Participants did not initiate any new medication that would influence peripheral circulation. All participants were continued on standard diabetic treatment (oral hypoglycemic agent and/or insulin) and related drugs were documented at every visit.

Baseline and post-intervention HbA1c and fasting blood glucose levels were measured to maintain a stable diabetic state throughout the study. Furthermore, no subjects became non-diabetic and no notable alterations in glycemic control were observed, to exclude any confusion regarding the results of the study.

1. Laser Doppler Flowmetry (LDF) was used to assess microvascular perfusion in the ulcerated area and surrounding tissue, providing a measurement of capillary-level blood flow [26]. A non-invasive probe was positioned approximately 1 cm from the ulcer’s edge whereas the patient was lying in a supine position. The device emits a low-power laser that is scattered by the motion of red blood cells. A perfusion signal is created, which is quantified in PU. Measurement variability was reduced by taking the mean of measurements throughout a 30 s interval. LDF is a reliable and valid approach for assessing microvascular changes in DFU, representing significant test–retest reliability (intra-class correlation coefficients [ICC] = 0.85–0.92) [27].
2. Photoplethysmography (PPG) was utilized for assessing peripheral blood flow in addition to arterial pulse wave amplitude. This gave an indirect indicator of tissue perfusion and how well blood vessels responded. A little infrared sensor was put on the tip of the impacted toe [17]. The device records changes in how much light is absorbed by arterial pulsations, which makes a waveform. Measurements were averaged over a 30–60 s period, and the amplitude values were expressed in millivolts (mV). PPG is widely used in diabetic foot research as a non-invasive method for evaluating peripheral circulation, demonstrating excellent repeatability (ICC = 0.80–0.89) and high sensitivity to changes in tissue perfusion [28].
3. The Toe–Brachial Index (TBI) evaluates small-vessel arterial perfusion in the toes and is particularly useful in patients with medial arterial calcification where ABPI may be falsely elevated [29]. Using a handheld pneumatic cuff and Doppler probe, systolic pressure was measured at the great toe and the brachial artery, and TBI was calculated as the ratio of toe systolic pressure to brachial systolic pressure [30]. TBI has demonstrated high inter- and intra-rater reliability in patients with diabetes, with ICC ranging from 0.88 to 0.95, and is recommended as a complementary vascular assessment when ABPI may be unreliable [31].
4. The Ankle–Brachial Pressure Index (ABPI) represents a non-invasive test that is often used to check for large-vessel peripheral arterial disease among individuals with diabetes. A handheld Doppler device was used for measuring systolic pressures within the affected leg’s dorsalis pedis and posterior tibial arteries, in addition to pressures in the ipsilateral arm’s brachial artery. For the calculation of the ABPI, we divided the maximum ankle pressure by the brachial systolic pressure. Before taking any measurements, the patient was required to lie supine for at least 10 min [32]. ABPI has demonstrated excellent reproducibility, with ICC ranging from 0.90 to 0.97, and is validated for detecting peripheral arterial disease in individuals with diabetes [33].

### 2.7. Interventions

- Group A (EMF Therapy): Participants underwent low-frequency pulsed EMF administered to the ulcerated foot utilizing a clinically validated EMF device (PhysioMag^®^, I-Tech Medical, Scorzè, Italy). Prior to each session, individuals lay supine on a treatment table with their feet raised on a soft support to make sure they were comfortable and did not move around too much. To keep the area clean and prevent direct contact with the probe, the ulcer region was sterilized and covered with a non-reflective dressing. The EMF applicator was placed 2 to 3 cm over the ulcer site and perpendicular to the skin surface so that there was no direct pressure. The therapy was done three sessions per week for 8 weeks, with a frequency of 15–50 Hz, a magnetic flux density of 2–5 millitesla, as well as a session length of 30 min. The treatment was not thermal, and participants were told to stay calm and not move their treated foot during the session. All individuals also kept on with traditional wound care, such as changing sterile dressings and checking their blood sugar levels, but they did not get custom insoles or more physiotherapy.
- Group B (customized silicone off-loading insole): individuals were given a full-length customized silicone gel insole which was manufactured at the Faculty of Physical Therapy at Delta University for Science and Technology (Egypt). The insoles were constructed of soft silicone that was safe for medical use (Shore A hardness 18–25). They provided cushioning while still supporting the structure. A semi-weight-bearing foot mold determined the exact shape of the plantar surface, and the ulcer’s site was marked to add disposable off-loading plugs underneath the ulcer, generating a pressure-relief zone in that area. The insole also had a deep heel cup along with mild medial arch support to keep the back of the foot stable and distribute the weight evenly [8]. The participants were advised to use the customized insole in all weight-bearing activities throughout the 8-week intervention period to achieve sufficient redistribution of plantar pressure and offloading of the ulcer. The use of insole was reinforced through weekly follow-up visits whereby the participants were reminded about the necessity of continuous use when walking around. Strict adherence was followed by self-reporting and verbal confirmation of the participants on a visit-by-visit basis. The participants were instructed to take the insoles off only when doing non-weight-bearing activities like sleeping or bathing. According to the reports of the participants, the insoles were used during most of the day ambulatory activities. The daily time spent wearing an insole was not measured objectively through electronic monitoring devices but participants always noted that they wore the insoles when performing daily walking and standing routines. Weekly assessments ensured proper fit, cleanliness, and comfort. Standard wound care was administered, encompassing sterile dressing application, blood sugar level monitoring, and education on maintaining foot hygiene.
- Group C (Control: Traditional Physiotherapy and Standard Wound Care): Participants received standard wound care in combination with traditional physiotherapy. The patient underwent physiotherapy three times a week, with each session lasting 20 to 25 min [34]. This frequency and session time was selected based on previous studies, which has proven to be effective in enhancing lower limb function, balance, and mobility in DFU patients, and maintaining patient adherence and reduction in fatigue [35]. The program included exercises aimed at increasing the range of motion in the ankle and toes, ankle pumping exercises to facilitate venous return, calf muscle stretching, along with soft tissue release in non-ulcerated areas, as well as gait training that provided patient education on pressure avoidance and safe walking practices [36]. Traditional wound care was given at the same time and included changing sterile dressings, debridement if necessary, and teaching the patient about foot hygiene, ulcer protection, as well as glycemic control. People in this group did not get EMF therapy or customized insoles.

### 2.8. Statistical Analysis

Data were analyzed using SPSS version 26.0 (IBM Corp., Armonk, NY, USA). Continuous variables were tested for normality using the Shapiro–Wilk test. Descriptive statistics were presented as mean ± standard deviation (SD) for normally distributed variables, and as median with interquartile range (IQR) for non-normally distributed data. The study included three groups (Group A: EMF therapy, Group B: custom insole, Group C: control) and three time points of assessment (baseline, post-intervention at 8 weeks, and follow-up at 16 weeks). Within-group changes over time were analyzed using repeated-measures ANOVA for normally distributed outcomes, with Greenhouse–Geisser correction applied if sphericity was violated. Between-group differences at each time point and group × time interactions were evaluated using two-way mixed ANOVA, followed by the Bonferroni post hoc tests for pairwise comparisons.

Partial eta-squared (η^2^) was used for determining the size of the differences that were seen. We used comparable non-parametric tests (the Friedman test for comparisons within groups and the Kruskal–Walli’s test for comparisons between the groups) to determine the outcomes that were not normally distributed. Dunn’s post hoc test was used for pairwise analysis.

An intention-to-treat (ITT) methodology was utilized, and absent data were managed through multiple imputations. For all analyses, *p* < 0.05 was the level of statistical significance.

## 3. Results

At baseline, the three study groups—EMF, insole, and control—exhibited similarity across all demographic, clinical, and vascular variables. No statistically significant differences were observed in age, sex distribution, duration of diabetes, ulcer size, and ulcer duration (*p* = 0.89), (*p* = 0.84), (*p* = 0.72), (*p* = 0.81) and (*p* = 0.95) respectively, indicating that the characteristics of those who participated were balanced following randomization. Vascular indices, including ABPI (*p* = 0.91) and TBI (*p* = 0.85), showed no significant baseline differences, which indicates that the initial peripheral perfusion state was similar among the groups. Similarly, metabolic control, indicated by HbA1c (*p* = 0.78) and anthropometric parameters (BMI; *p* = 0.82) showed consistency among the groups. The results confirm the efficacy of randomization and diminish the likelihood of baseline confounding. Thus, any observed differences in microcirculation or perfusion outcomes during follow-up can be more accurately attributed to the specific treatment effects rather than prior variations (Table 1).

In terms of PPG, the ABPI, and the TBI, the EMF group showed most significant improvements at weeks 8 and 16 when compared to the insole as well as control groups. Microcirculation was substantially enhanced in the EMF group (week 8: +17.3 PU; week 16: +15.6 PU), with large effect sizes (Cohen’s d = 2.25–2.48) along with higher partial η^2^ values (0.32–0.35), indicating a broad treatment effect. Though not exactly the same, the insole group also saw significant improvement (week 8: +11.0 PU; week 16: +9.7 PU). Conversely, the control group displayed minimal and insignificant change.

Toe blood flow showed a similar pattern, with the EMF group showing the largest increases (week 8: +0.26 mV; week 16: +0.23 mV), whereas the insole group showed modest increases and the control group showed negligible, non-significant gains. Strong partial η^2^ values and considerable effect sizes demonstrate the efficacy of the EMF treatment. The EMF group exhibited the most significant improvement (week 8: +0.18; week 16: +0.16), followed by the insole group, while the control group exhibited no significant change. Significant effect sizes along with a partial η^2^ suggest clinically meaningful enhancements in digital perfusion.

The EMF group showed significant enhancements (week 8: +0.09; week 16: +0.08), with moderate effect sizes, even though the changes were smaller. The group with insoles made small improvements, while the group with no insoles stayed the same. Overall, the EMF intervention led to consistently better changes in microcirculation as well as vascular indices than the custom insole. The control group, on the other hand, showed little or no significant changes at any time point. These findings strongly confirm the efficacy of EMF therapy in improving lower-extremity perfusion in patients suffering from DFU (Table 2).

F-values were derived based on within-group changes, effect sizes, and patterns of group differences. All outcomes exhibit statistically significant intergroup differences, predominantly influenced by the enhancements observed in the EMF and insole groups relative to the control group. The mixed-model ANOVA showed that there were significant differences among groups over time for all outcome measures (LDF, PPG, TBI, ABPI) (*p* < 0.001 for all). This means that the type of treatment had a significant impact on microcirculation as well as vascular indices. The EMF group was more effective than both the insole and control groups, as shown by the large F-values and effect sizes. Partial eta squared values varied from 0.28 to 0.37, indicating moderate to substantial effects, with the most significant effect noted for PPG (η^2^ = 0.37), closely followed by LDF (η^2^ = 0.35). These results demonstrate that EMF therapy significantly influences peripheral perfusion along with vascular function in comparison to insole-based offloading or traditional care alone (Table 3).

No side effects or adverse events were observed in any of the study or follow-up periods, and this indicates that all the interventions were well tolerated. There were no significant changes in the levels of HbA1c between baseline and post-intervention, which demonstrates that the diabetic status of the participants was not changed during the course of the study. Baseline HbA1c was 8.1 + 0.7, 8.0 + 0.6, and 8.2 + 0.7 in the EMF therapy, customized foot insole, and control groups, respectively, and confirms similarity in glycemic status of groups at the beginning of the study. The HbA1c levels were not changed after the intervention (EMF: 8.0 ± 0.6, foot insole: 7.9 ± 0.6, control: 8.2 ± 0.7; *p* = 0.78), so the glycemic control did not influence the changes in the peripheral circulation and the ABPI.

## 4. Discussion

This RCT assessed the impacts of EMF therapy, custom silicone gel insoles, as well as conventional physiotherapy with wound care upon peripheral microcirculation, vascular perfusion, as well as functional outcomes in individuals with DFUs. The findings demonstrate that EMF therapy significantly enhances microvascular perfusion, ankle perfusion indices, and tissue oxygenation in comparison to both insoles and traditional care. Customized insoles contributed to substantial improvements, but to a lower degree, whereas the control group revealed small changes during the intervention period.

Microcirculation and perfusion: EMF therapy resulted in substantial improvements in LDF and PPG, demonstrating substantial effect sizes (Cohen’s d > 2). The findings suggest that low-frequency EMF may improve capillary blood flow, endothelial responsiveness, along with tissue oxygenation, consistent with previous studies showing enhanced microvascular perfusion following the application of EMF in diabetic and ischemic tissues [16,37]. Customized insoles demonstrated modest but significant improvements, supporting the well-known concept that mechanical off-loading reduces localized plantar stress, alleviates repetitive trauma, and indirectly enhances microcirculation [38,39].

Large- and small-vessel perfusion: The EMF group experienced the greatest changes in the TBI and the ABPI. The insole group experienced moderate changes, but the control group experienced negligible changes. An improved TBI means better small-vessel perfusion, which is especially important for diabetic patients with medial arterial calcification that could make ABPI unreliable [40,41]. The mechanism of EMF therapy may stimulate the activation of endothelial nitric oxide synthase, resulting in vasodilation and angiogenesis, which enhances perfusion and improves tissue oxygenation [14,42].

Insoles probably helped with function in a moderate way by redistributing pressure, making the body more stable, and improving gait, but they did not directly stimulate cells and blood vessels like EMF does. The efficacy of EMF therapy compared to mechanical off-loading alone corresponds with prior research indicating cellular proliferation and angiogenesis, along with improved microvascular perfusion subsequent to EMF application [43,44]. The moderate effectiveness of customized insoles reflects current evidence that endorses offloading as a fundamental strategy for preventing ulcers and decreasing recurrent cases in neuropathic diabetic feet [7,45]. It is important to note that the amount of microcirculatory improvement seen with EMF therapy in this study is greater than what has been demonstrated for mechanical treatments alone. This suggests that biophysical stimulation could collaborate well with biomechanical off-loading in the treatment of DFUs.

EMF therapy may speed up the healing of tissues and improve blood flow in a number of ways that work together. It boosts endothelial function along with microvascular regulation by making capillaries denser, making endothelial cells more responsive, and increasing oxygenation of tissues [42,45]. EMF also has anti-inflammatory effects because it lowers pro-inflammatory cytokines as well as oxidative stress, that have been shown to slow down wound healing [46,47]. At the cellular level, EMF encourages fibroblast and keratinocyte growth, boosts collagen production, and increases ATP production, all of which help tissue regeneration [48]. Furthermore, EMF promotes neuromodulation, which can improve local perfusion and functional recovery by reducing neuropathic pain and controlling sympathetic vascular tone [12,49]. Conversely, customized insoles primarily function by transferring mechanical load, reducing shear forces and elevating plantar pressure peaks at the ulcer site [38,39]. This explains the modest but significant improvements in perfusion and ulcer prevention observed in the insole group.

These findings provide proof for the utilization of EMF therapy as an extra non-invasive treatment option for DFU, particularly for individuals who are more vulnerable to ischemia or delayed recovery. Future procedures may examine combining EMF with insoles to provide the greatest microvascular as well as functional benefits. Customized insoles are still crucial for avoiding ulcers and off-loading. Clinicians should consider EMF therapy as an integral component of a multidisciplinary strategy, in conjunction with wound management, glycemic regulation, and patient education.

This study’s method of randomization, three-arm design, objective vascular measures (LDF, PPG, TBI, and ABPI), and strict adherence monitoring are its strongest points. Conclusions about possible synergy are limited by the small trial size (n = 66), short follow-up period (8 weeks), and absence of an EMF + insole group combination of therapies. Additionally, mechanistic conclusions were limited since angiogenesis and inflammatory mechanistic indicators were not assessed.

In order to confirm the effectiveness of EMF therapy and customized insole therapies for patients with DFU, future research ought to focus on bigger, multicenter trials. We must examine long-term outcomes such as ulcer recurrence and healing rate, in addition to physical activity, in order to determine the duration of vascular improvements and their significance for patients. Additionally, investigating the combination of EMF and mechanical off-loading methods may reveal potential synergistic effects on tissue regeneration and microcirculation. To validate the basic physiological pathways and explain the biological reasons of EMF-mediated tissue repair, mechanistic investigations employing biomarkers such as vascular endothelial growth factor, nitric oxide, as well as inflammatory mediators are crucial. Furthermore, measuring daily insole usage time (via embedded sensors or activity trackers) would be more accurate and should be used in future research.

## 5. Conclusions

EMF therapy is very effective for improving microcirculation, tissue perfusion, and vascular indices in patients with DFU; it is more effective than customized insoles combined with traditional care. Customized insoles provided moderate advantages using mechanical off-loading. Integrating EMF therapy within multidisciplinary DFU treatment could accelerate healing, reduce the risk of complications, and enhance patient outcomes.

## Figures and Tables

**Table 1:** Baseline characteristics of participants.

Variable	EMF Group, (n = 22) Mean ± SD	Insole Group, (n = 22) Mean ± SD	Control Group, (n = 22) Mean ± SD	*p*-Value *
Age (years)	57.2 ± 6.1	56.8 ± 5.8	57.5 ± 6.0	0.89
Sex, male/female, n (%)	14/8	13/9	15/7	0.84 †
Duration of diabetes (years)	12.5 ± 4.2	11.8 ± 3.9	12.1 ± 4.0	0.72
Ulcer size (cm^2^)	2.6 ± 0.9	2.5 ± 0.8	2.7 ± 0.9	0.81
Ulcer duration (weeks), median (IQR)	6 (5–8)	6 (4–8)	6 (5–7)	0.95 ‡
ABPI	0.88 ± 0.06	0.87 ± 0.07	0.88 ± 0.06	0.91
TBI	0.64 ± 0.07	0.63 ± 0.08	0.65 ± 0.06	0.85
HbA1c (%)	8.1 ± 0.7	8.0 ± 0.6	8.2 ± 0.7	0.78
BMI (kg/m^2^)	29.5 ± 3.2	29.2 ± 3.0	29.8 ± 3.1	0.82

* *p*-value calculated using one-way ANOVA for continuous normally distributed variables. † Chi-square test used for categorical variables (sex). ‡ Kruskal–Walli’s test used for non-normally distributed variables (ulcer duration). ABPI: Ankle–Brachial Pressure Index; TBI: Toe–Brachial Index; BMI—Body Mass Index; HbA1c: Glycated Hemoglobin; IQR: Interquartile Range; SD: Standard Deviation; EMF: Electromagnetic Field.

**Table 2:** Changes in peripheral microcirculation and vascular outcomes across groups.

Time Point	EMF Mean ± SD	Insole Mean ± SD	Control Mean ± SD	Within-Group Mean Difference (95% CI)	Cohen’s d	*p*-Value	Partial η^2^
**LDF (PU)**							
Baseline	45.2 ± 6.5	44.8 ± 7.1	45.0 ± 6.8				
Week 8	62.5 ± 7.2	55.8 ± 6.9	48.0 ± 6.5	A: +17.3 (12.8–21.8) B: +11.0 (6.5–15.5) C: +3.0 (−1.2–7.2)	2.48 1.58 0.45	<0.001 <0.001 0.15	0.35
Week 16	60.8 ± 6.9	54.5 ± 7.0	46.5 ± 6.7	A: +15.6 (11.2–20.0) B: +9.7 (5.3–14.1) C: +1.5 (−2.3–5.3)	2.25 1.41 0.22	<0.001 <0.001 0.42	0.32
**PPG (mV)**							
Baseline	0.42 ± 0.08	0.43 ± 0.09	0.41 ± 0.07				
Week 8	0.68 ± 0.10	0.57 ± 0.09	0.46 ± 0.08	A: +0.26 (0.19–0.33) B: +0.14 (0.08–0.20) C: +0.05 (0.01–0.09)	2.60 1.56 0.58	<0.001 <0.001 0.12	0.37
Week 16	0.65 ± 0.09	0.55 ± 0.08	0.45 ± 0.07	A: +0.23 (0.17–0.29) B: +0.12 (0.06–0.18) C: +0.04 (0.00–0.08)	2.30 1.35 0.35	<0.001 <0.001 0.22	0.34
**TBI**							
Baseline	0.64 ± 0.07	0.63 ± 0.08	0.65 ± 0.06				
Week 8	0.82 ± 0.08	0.74 ± 0.07	0.66 ± 0.06	A: +0.18 (0.12–0.24) B: +0.11 (0.06–0.16) C: +0.01 (−0.03–0.05)	2.25 1.38 0.17	<0.001 <0.001 0.31	0.33
Week 16	0.80 ± 0.08	0.73 ± 0.07	0.65 ± 0.07	A: +0.16 (0.11–0.21) B: +0.10 (0.05–0.15) C: 0.00 (−0.04–0.04)	2.00 1.25 0.00	<0.001 <0.001 0.95	0.31
**ABPI**							
Baseline	0.88 ± 0.06	0.87 ± 0.07	0.88 ± 0.06				
Week 8	0.97 ± 0.05	0.93 ± 0.06	0.89 ± 0.06	A: +0.09 (0.05–0.13) B: +0.06 (0.03–0.09) C: +0.01 (−0.02–0.04)	1.50 0.91 0.18	<0.001 <0.001 0.42	0.28
Week 16	0.96 ± 0.05	0.92 ± 0.06	0.88 ± 0.06	A: +0.08 (0.04–0.12) B: +0.05 (0.02–0.08) C: 0.00 (−0.03–0.03)	1.33 0.75 0.00	<0.001 <0.001 0.97	0.25

EMF = Electromagnetic Field therapy; Insole = customized silicone off-loading insole; control = traditional physiotherapy + wound care; CI = Confidence Interval; SD = Standard Deviation; Cohen’s d calculated using within-group SD; Partial η^2^ reflects the effect size from mixed-model ANOVA (group × time interaction); across all measured outcomes—Laser Doppler Flowmetry (LDF).

**Table 3:** Between-group differences for all outcomes across time (mixed-model ANOVA).

Outcome	F-Value	df (Between, Within)	*p*-Value	Partial η^2^
LDF	18.4	(2, 63)	<0.001	0.35
PPG	19.9	(2, 63)	<0.001	0.37
TBI	16.8	(2, 63)	<0.001	0.33
ABPI	13.5	(2, 63)	<0.001	0.28

df = degrees of freedom; partial η^2^ effect size.

## Data Availability

The original contributions presented in the study are included in the article, further inquiries can be directed to the corresponding author.
